# Diagnostic decisions of specialist optometrists exposed to ambiguous deep-learning outputs

**DOI:** 10.1038/s41598-024-55410-0

**Published:** 2024-03-21

**Authors:** Josie Carmichael, Enrico Costanza, Ann Blandford, Robbert Struyven, Pearse A. Keane, Konstantinos Balaskas

**Affiliations:** 1 University College London Interaction Centre (UCLIC), UCL, London, UK; 2 Institute of Ophthalmology, NIHR Biomedical Research Centre at Moorfields Eye Hospital NHS Foundation Trust and UCL, London, UK

**Keywords:** Ophthalmology, OCT, Retinal disease, Artificial intelligence, Human–computer interaction, Diagnosis, Medical imaging, Eye diseases, Diagnostic markers

## Abstract

Artificial intelligence (AI) has great potential in ophthalmology. We investigated how ambiguous outputs from an AI diagnostic support system (AI-DSS) affected diagnostic responses from optometrists when assessing cases of suspected retinal disease. Thirty optometrists (15 more experienced, 15 less) assessed 30 clinical cases. For ten, participants saw an optical coherence tomography (OCT) scan, basic clinical information and retinal photography (‘no AI’). For another ten, they were also given AI-generated OCT-based probabilistic diagnoses (‘AI diagnosis’); and for ten, both AI-diagnosis and AI-generated OCT segmentations (‘AI diagnosis + segmentation’) were provided. Cases were matched across the three types of presentation and were selected to include 40% ambiguous and 20% incorrect AI outputs. Optometrist diagnostic agreement with the predefined reference standard was lowest for ‘AI diagnosis + segmentation’ (204/300, 68%) compared to ‘AI diagnosis’ (224/300, 75% p = 0.010), and ‘no Al’ (242/300, 81%, p =  < 0.001). Agreement with AI diagnosis consistent with the reference standard decreased (174/210 vs 199/210, p = 0.003), but participants trusted the AI more (p = 0.029) with segmentations. Practitioner experience did not affect diagnostic responses (p = 0.24). More experienced participants were more confident (p = 0.012) and trusted the AI less (p = 0.038). Our findings also highlight issues around reference standard definition.

## Introduction

Artificial intelligence (AI) powered technologies are rapidly evolving in the medical domain and show promise across a range of clinical applications^1^. For example, in medical imaging, AI can detect malignancy in breast tissue during mammography^2^ and pre-cancerous polyps during colonoscopy^3^. Additionally, it has displayed impressive performance for distinguishing between diagnoses using multi-class outputs in skin lesion classification^4^ and ophthalmic disease^5^.

Ophthalmology is at the forefront of such digital translation^6^. In 2018 the FDA approved the first autonomous AI medical diagnostic system for detecting more than mild diabetic retinopathy^7^. In 2019/2020, ophthalmic departments in the UK had 7.9 million outpatient attendances^8^ the highest number of any medical specialty within the NHS. With the ubiquitous use of modern ophthalmic imaging for eye disease diagnosis and management, and the low number of ophthalmologists per capita, ophthalmic services may benefit from deployment of AI decision support systems (AI-DSS) to help cope with demand.

Clinical AI systems have shown human expert-level performance for several ophthalmic use-cases, yet little is known about how clinicians might engage with them in practice^1,9^. Recent studies involving suspected breast cancer^10^ and skin cancer^11^ have reported a strong preference for a symbiotic relationship between clinicians and AI, over fully autonomous AI systems, and many clinicians regard the prospect of AI systems with scepticism and resistance^1^. Increased 'explainability' of AI systems has been proposed to enhance algorithmic transparency and user confidence, though currently used methods such as saliency maps^12^ have not achieved this, mainly due to their post-hoc design.

Minimal human–computer interaction (HCI) research has focused on AI in healthcare. One important aspect of human-AI interaction is whether clinicians' decisions are affected by displaying automated outputs. This has been investigated for various healthcare specialities^13–18^ and factors affecting how clinicians are influenced by such outputs have included user experience, user confidence and cognitive style^19–22^. However, most previous research has focussed on non-AI systems.

In this study, we used outputs from an ophthalmic AI-DSS^5,23^ to investigate whether clinicians' diagnostic decisions were influenced by displaying deliberately selected ambiguous/incorrect AI outputs, from the original validation cohort^5^. The system performs automated diagnosis of retinal disease and comprises two AI algorithms which analyse retinal optical coherence tomography (OCT) scans to produce segmentation maps along with multi-class outputs for diagnostic suggestions. The segmentation algorithm highlights and quantifies pathological features on OCT images using a colour coded overlay aligned over each OCT image (Supplementary Fig. 1). The classification algorithm then analyses the segmentations to provide multi-class diagnosis outputs and a referral suggestion. The system offers the opportunity to elicit distinct elements of Human-AI Interaction and their differential effect on diagnostic decisions. The segmentation algorithm, for example, could conceivably help users to better understand the recommendations made by the classification algorithm by highlighting the OCT features detected as pathological, hence informing the classification decision.

In this study, we used quantitative methods to assess the effect of this AI-DSS on the interpretation of OCT scans by trained optometrists. Although rare, we chose to focus on cases where the AI diagnostic outputs were incorrect (disagreed with the reference standard) or ambiguous (more than one diagnosis proposed with high probability), to explore how users may interact with AI outputs when they fall into one of two rare occurrences: (a) They are truly incorrect, (b) There is true clinical ambiguity about the correct diagnosis. A third occurrence emerged through a post hoc analysis of cases with reduced agreement: AI outputs that occasionally appear incorrect against an imperfect reference standard. We explored whether diagnostic decisions were influenced by the type of AI outputs displayed (diagnostic classification alone or with segmentation overlay). Level of trust in the AI outputs was also assessed.

## Methods

### Study overview

**Figure 1:**
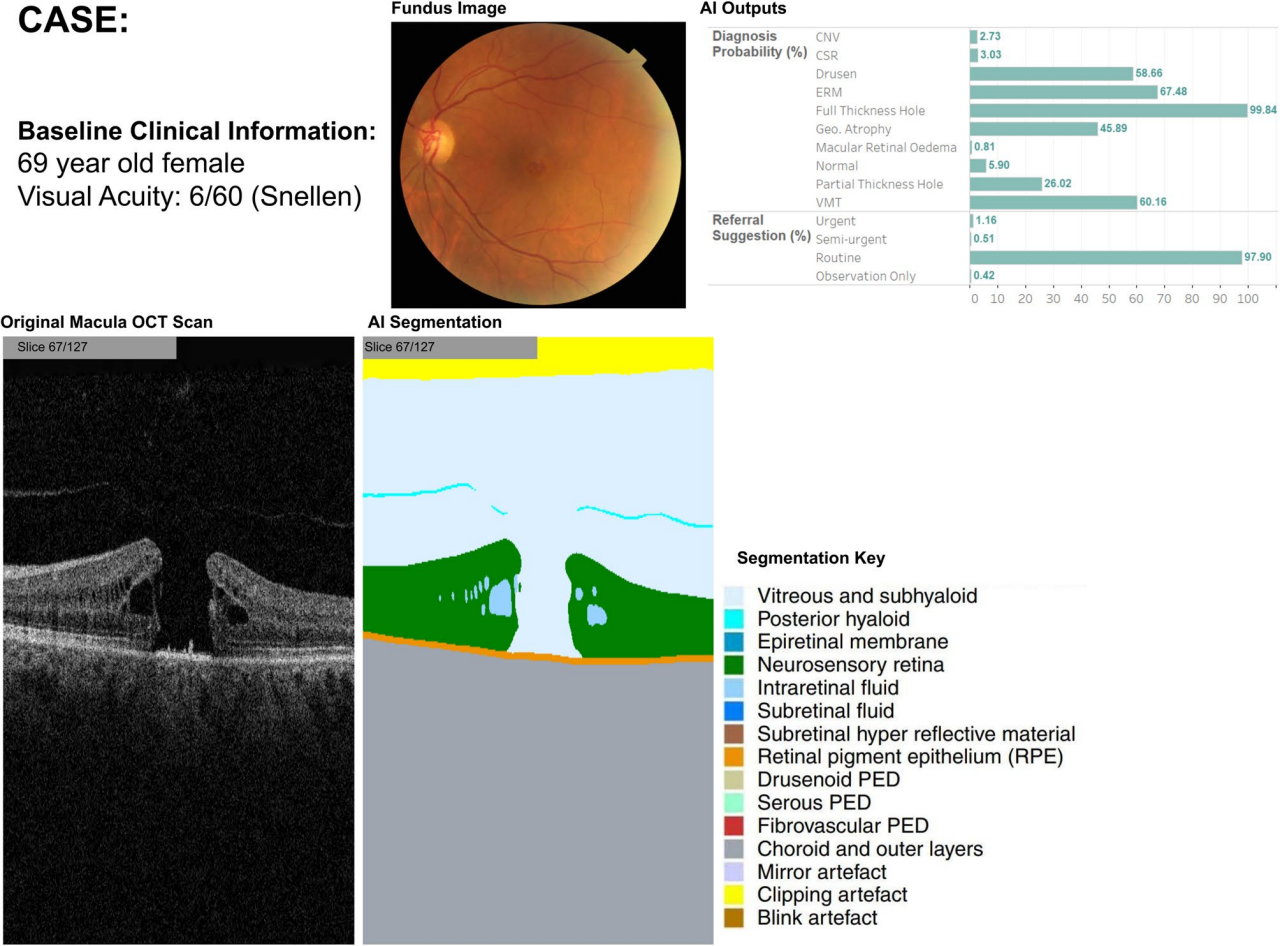
The different elements presented during review of clinical cases. This example represents a case in which baseline information, AI diagnosis suggestions and segmentation overlays are all presented. For other cases, only elements of this example would be presented (i.e., only the AI diagnoses).

Thirty clinical cases were assessed by 30 optometrists. For each case, participants were asked to choose the single most probable retinal diagnosis from ten options. They also chose their referral decision from four options (Fig. 1) and indicated their confidence in their decision using a 5-point Likert scale. We focused the primary analysis on comparing optometrists' diagnostic decisions to the 'reference standard' clinical diagnosis for each case, as referral decisions post-diagnosis can be context-dependent (e.g., healthcare system, departmental protocols). The number of cases was limited by the effort and time the study required of participants, especially as it relied on clinicians participating in their own time without any incentive. We considered 30 cases per participant as the maximum time we could request of them, estimating it would take them around 40–50 min (if they engaged in the assessment continuously).

For 10 cases (‘no AI’), participants were provided with baseline information that included demographic and clinical characteristics (age, visual acuity, and biological sex), a colour retinal photograph and a full-volume macular OCT scan consisting of 128 B-scans or ‘slices’ (Fig. 1). All OCT imaging was acquired using the Topcon 3D OCT-2000. We did not explore the potential variability that might arise from using different OCT devices. Participants were able to ‘scroll’ through the 128 images using their arrow keys, allowing them to pause on any slices of interest. This closely mimicked their method of scrolling through macular OCT scans in real-world practice. A separate 10 cases were presented with baseline information plus the raw AI outputs for diagnostic classifications and referral probability (as a horizontal bar chart) (‘AI diagnosis’). A further 10 cases were presented with baseline information, the diagnostic classification output and, additionally, the segmentation output of the AI algorithm—i.e., a colour-coded overlay highlighting clinical features within each of the OCT 128 B-scans (‘AI diagnosis + segmentation’). This segmentation output was scrolled through sequentially with the corresponding 128 OCT slices. The methods of displaying the raw outputs from the model were based on a mock visualisation used in the original validation paper which has not been validated as an optimal method of displaying outputs. This visualisation consisted of an average segmentation map calculated from the results of five hypotheses from a segmentation network. We chose to include two different types of presentation format (i.e., AI support with and without segmentation maps) as we believed these two formats may affect diagnostic decisions differently. Diagnostic outputs encompass a constellation of potential imaging features on OCT that should and/or could be present to inform the clinical diagnosis. A segmentation output highlights the presence or absence of specific pathological imaging features on OCT (which feed into the diagnostic model to inform its prediction), but these features could be present in more than one retinal diagnoses. After completing each set of 10 cases with AI information (‘AI diagnosis’ and ‘AI diagnosis + segmentation’), participants recorded their level of trust in the AI outputs using a 5-point Likert scale.

The research adhered to the tenets of the Declaration of Helsinki. All patient information, images and scans were used in line with Research Ethics Committee (REC) approval (20/HRA/2158). Data acquired from study participants was in line with UCL interaction centre Research Ethics Committee approval (UCLIC/1819/006/BlandfordProgrammeEthics).

### Choice of cases

The 30 cases used data and AI analysis generated as part of a published study^5^. The original validation dataset comprised anonymized scans from n = 997 patients with a range of retinal diseases who attended Moorfields Eye Hospital (MEH) between 1 June 2012 and 31 January 2017. Images with poor quality and/or significantly reduced signal strength were excluded.

Cases were chosen by JC to cover a range of macular pathologies and to include healthy scans (Supplementary Table 4). When choosing cases, the diagnoses suggested by the AI were compared to the 'reference standard' clinical diagnosis, decided by an ophthalmologist during a face-to-face examination. The cases were matched across the three presentations to participating optometrists with respect to 'reference standard' diagnosis and difficulty. The cases were purposely chosen to include a disproportionately large number of instances where the AI disagreed with the ‘reference standard’ (20% of cases) or was ambiguous (40% of cases) as we wanted to focus on interesting cases whereby incorrect/ambiguous AI may influence participants' decisions and were aiming to inflate the number of incorrect/ambiguous outputs while retaining some resemblance to a real-life case-mix. Fifty per cent of cases were determined by a consultant ophthalmologist and medical retina (MR) specialist (KB) as also being truly clinically ambiguous based on the OCT findings. The remaining 40% of cases were considered unambiguous with the AI diagnosis agreeing with the 'reference standard'. The actual incidence of cases where the AI diagnosis disagrees with the reference standard or provides uncertain outputs is much smaller than in our study. When assessing the sensitivity and specificity of the AI diagnosis for all assessed conditions, using receiver operating characteristic curve (ROC) diagrams, the area under the curve (AUC) was reported as between 96.63 (for epi-retinal membrane) and 100.00 (for full-thickness macular hole) in the original validation study of the AI-DSS^5^. No information about AI accuracy was provided to participants until debriefing.

### Study set up

An online survey tool was used for submitting responses. A HTML case viewing interface (Fig. 1) was accessible only by study participants and investigators within the MEH network. Basic training about the AI segmentation overlays and diagnostic outputs was provided to ensure all participants had a similar level of understanding (Supplementary methods).

### Participants

Thirty qualified optometrists were recruited; all worked at MEH and none had previous exposure to the AI-DSS. Half of the participants were recruited to fit predetermined criteria of 'more experienced', and half 'less experienced'. These group allocation criteria were decided with an MR specialist (KB), based on experience in an MR clinic, which was used as a surrogate for familiarity with interpreting OCT scans. No minimum number of years' experience was required. Informed consent was obtained from participants via an online form prior to beginning the survey.

**Figure 2:**
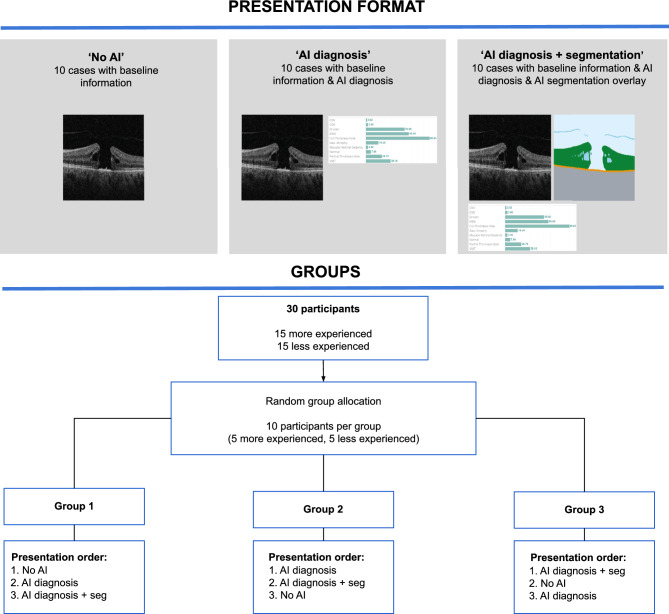
Order of Case Presentation. Participants were randomly assigned to one of three groups. Each group viewed the clinical cases in a different order to account for possible order effects on responses and contained five more experienced participants and five less experienced participants.

Each participant was randomly allocated to one of three groups, with each group experiencing all three presentation formats in a different order (balanced through a Latin square). This counterbalanced order was to control for presentation order as a possible confounding factor influencing results (Fig. 2). Each group contained five more experienced optometrists and five less experienced ones. All 30 optometrists saw each of the 30 cases.

### Statistics

Quantitative analysis was conducted in SPSS for Windows version 28 (SPSS Inc, Chicago, IL, USA) and the Windows aligned rank transform (ART) open-source application^24^. ANOVA was used to test for a significant difference between categorical groups post ART adjustment**.** A p value of < 0.05 was considered statistically significant.

## Results

### Diagnostic responses

Each of the 30 participants answered diagnostic questions for 30 cases, resulting in 900 responses in total. The median completion time taken to complete the 30 cases was 44 min, 50 s. Completion time varied widely between participants (range: 16 min, 29 s to 182 min, 56 s), suggesting some participants completed the study whilst multitasking. Indeed, prior work pointed out that multitasking is common for participants of online studies^25^. Thus, further analysis of task completion time would be of limited value. An ANOVA with ART adjustment revealed significant differences in reference standard-aligned responses across the three presentation formats (p < 0.001) (Table 1). A borderline effect of the order of case presentation was also found (p = 0.049). There was no significant effect of experience on the number of reference standard-aligned responses. When testing interactions between reference standard-aligned responses and potential confounding factors, a significant interaction with order and presentation format was found. All other interactions showed no significant effect.

**Table 1:** Results from ANOVA testing on number of diagnoses in agreement with the reference standard.

Factor(s)	Diagnosis	
	F-value	p-value
1 Experience	1.426	0.244
2 Order	3.195	0.049*
3 Presentation format	15.036	< 0.001*
4 Experience: Order	2.046	0.140
5 Experience: Presentation	1.877	0.164
6 Order: Presentation	2.903	0.032*
7 Experience:Order:Presentation	1.400	0.280

ANOVA performed on results using aligned rank transform (ART). Results for factors 1–3 represent the effect of a single factor on diagnosis. Results for factors 4–7 represent the effect of two or more factors interacting. Values in bold represent statistically significant results.

*p values considered statistically significant.

### Effect of presentation format

The participants’ responses were divided into 3 classes, based on the presentation format. In the ‘no AI’ group, 242/300 (81%) responses agreed with the reference standard. In the ‘AI diagnosis’ group, 224/300 (75%) agreed with the reference standard. In the ‘AI diagnosis + segmentation’ group, 204/300 (68%) agreed with the reference standard. Significant differences in responses agreeing with the reference standard were found between all 3 pairs: ‘no AI’ vs ‘AI diagnosis’ (p = 0.049) [became non-significant when excluding the results from the 3 cases of Epiretinal Membrane (ERM). See supplementary material], ‘no AI’ vs ‘AI diagnosis + segmentation’ (p < 0.001) and ‘AI diagnosis + segmentation’ vs ‘AI diagnosis’ (p = 0.011).

### Effect of case order

A post-hoc assessment within groups (Fig. 2) revealed a significantly higher number of responses agreeing with the reference standard when comparing the first set of 10 cases viewed vs the third (p = 0.041). No significant differences were found between the first set of 10 cases viewed vs the second (p = 0.771) or the second vs the third (p = 0.514).

### Interaction between presentation format and case order

**Figure 3:**
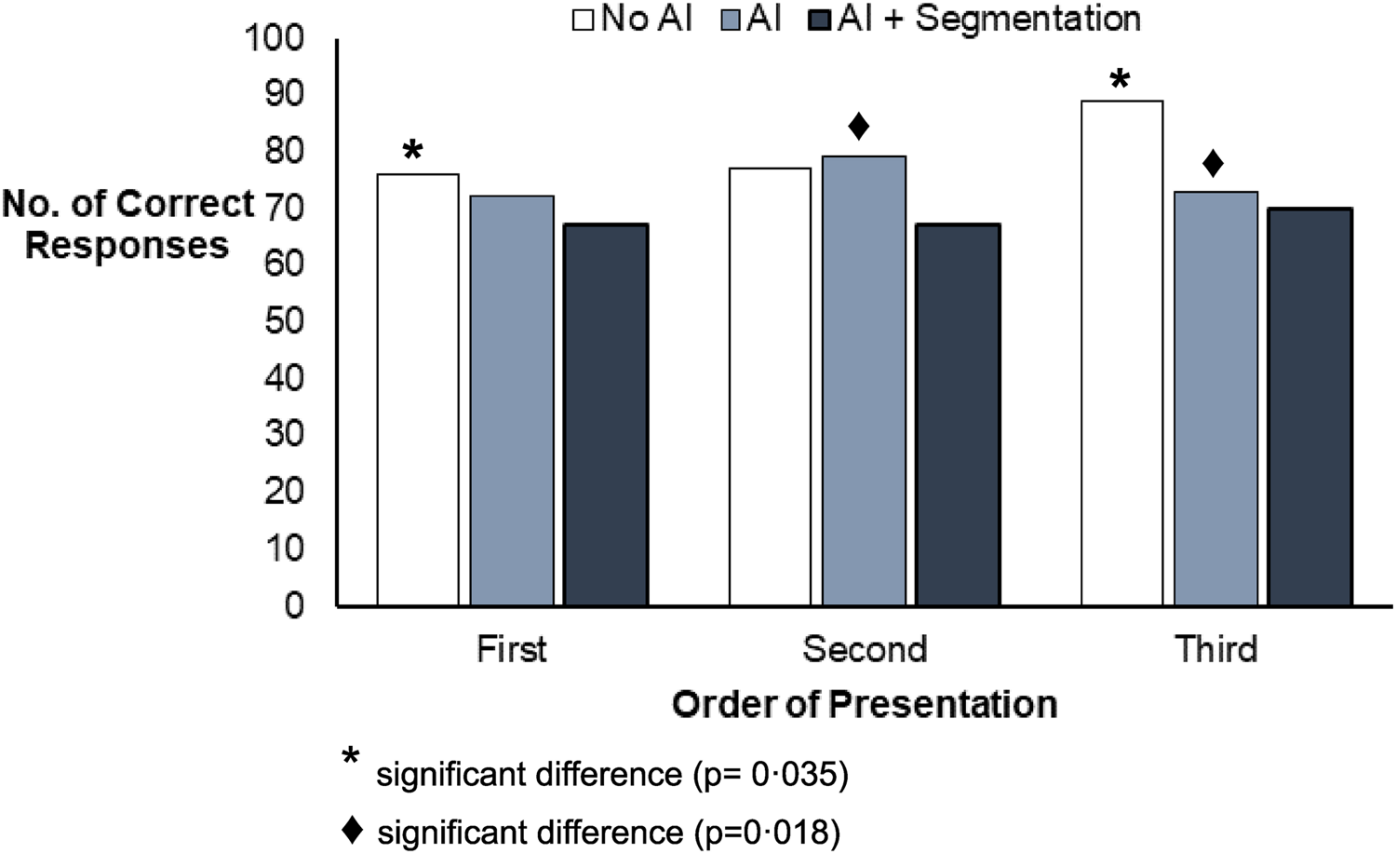
Number of ‘correct’ diagnostic responses (in agreement with a pre-defined reference standard diagnosis) for three presentation formats, based on the order they were viewed by participants. Post-hoc comparisons were carried out for the presentation formats.

When making post-hoc comparisons (Fig. 3), there was a significant difference in responses agreeing with the reference standard between ‘no AI’ presentation viewed first vs third (p = 0.035) and between ‘AI diagnosis’ presentation viewed second vs third (p = 0.018). No other comparisons were significant.

### Participants’ level of agreement with AI

When assessing agreement with AI outputs, there was a significant effect of presentation format (p = 0.001) (Table 2). There was no significant effect of experience (p = 0.080) or presentation order (p = 0.816) and no significant interactions.

**Table 2:** Results from ANOVA testing on number of responses in agreement with AI outputs.

Factor(s)	Agreement	
	F-value	p-value
1 Experience	0.065	0.080
2 Order	0.216	0.816
3 Presentation Format	11.890	0.001*
4 Experience: Order	1.148	0.326
5 Experience:Presentation	0.790	0.391
6 Order:Presentation	0.260	0.772
7 Experience:Order:Presentation	1.058	0.355

ANOVA performed on results using aligned rank transform (ART). Results for factors 1–3 represent the effect of a single factor on agreement with AI. Results for factors 4–7 represent the effect of two or more factors interacting. Values in bold represent statistically significant results. *p-value statistically significant.

*p values considered statistically significant.

### Effect of presentation format on agreement with AI

To compare the level of agreement with 'correct' AI diagnosis for responses given with and without segmentations, we divided responses into four groups, based on the participant being ‘correct’/’incorrect’ and the AI being ‘correct’/’incorrect’. For the 70% of cases where the AI diagnosis agreed with the reference standard, an ANOVA with ART correction revealed that participants agreed with the AI diagnosis significantly more when segmentation was not displayed (p < 0.001, Table 3). In contrast, for cases where AI diagnosis disagreed with the reference standard (30%) no significant effect of segmentation display on agreement with AI diagnosis was found (p = 0.236).

**Table 3:** Total participant responses for diagnostic decisions divided into four categories based on being ‘correct’/’incorrect’ and in relation to AI diagnosis being ‘correct’/’incorrect’.

A) AI Diagnosis			
	AI Correct	AI Incorrect	
Participant Correct	**199 (66%)**	25 (8%)	Total 224(75%)
Participant Incorrect	**11 (4%)**	*65 (22%)	Total 76(25%)
	Total 210(70%)	Total 90(30%)	

B) AI Diagnosis + Segmentation			
	AI Correct	AI Incorrect	
Participant Correct	**174 (58%)**	30 (10%)	Total 204(68%)
Participant Incorrect	**36 (12%)**	**60 (20%)	Total 96(32%)
	Total 210(70%)	Total 90(30%)	

(A) represents the responses provided for cases where AI diagnoses were displayed (N = 300). (B) represents the responses provided for cases where AI diagnosis plus segmentation overlays were displayed (N = 300). Numbers highlighted in bold represent a significant difference in ‘correct’ participant responses between (A) and (B) (p < 0.001 with ART and ANOVA analysis).

*In 58/65 incorrect responses, the participant and AI gave the same ‘incorrect’ diagnosis.

**In 53/60 incorrect responses, the participant and AI gave the same ‘incorrect’ diagnosis.

### Case analysis

To explore the reduced agreement with AI diagnosis when segmentation overlays were displayed, we completed a post-hoc analysis by assessing matched cases, with respect to diagnosis and difficulty, across the presentation formats, and identified two distinct sets with an obvious difference in responses between the ‘AI diagnosis + segmentation’ and the other two presentation formats. The following two examples are particularly informative.

#### Set 1

**Figure 4:**
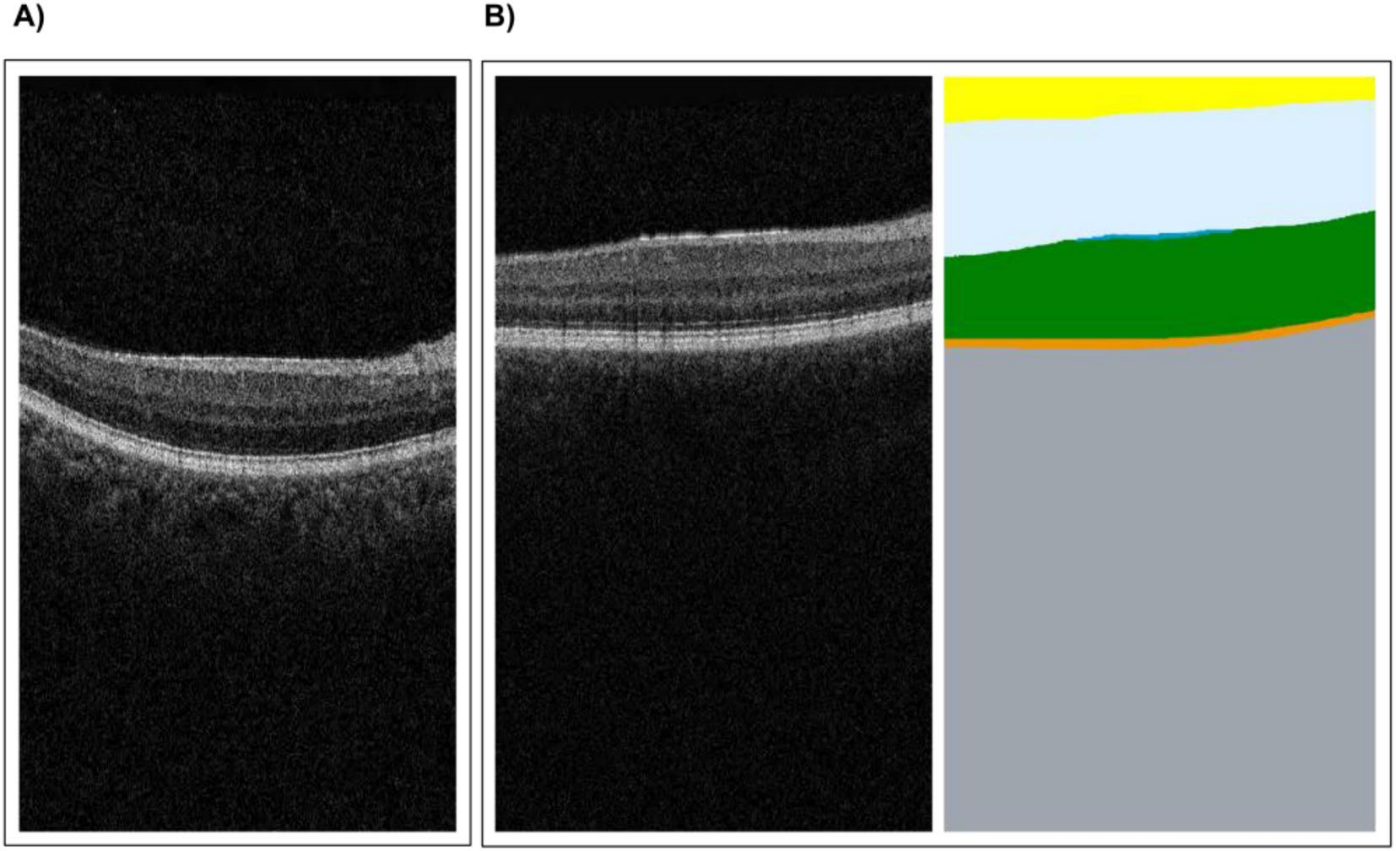
One image taken from two matched OCT scans. (**A**) OCT presented with AI diagnosis. (**B**) OCT presented with AI diagnosis plus segmentation. Very similar areas of hyper-reflectivity are present, which for (**B**) was identified as an epiretinal membrane (ERM) by the segmentation overlay (dark blue area). Both (**A**) and (**B**) were classified as normal by the AI diagnosis.

For set one, the reference standard and AI diagnosis was 'normal', which 29 and 28 participants agreed with in the ‘AI diagnosis’ and ‘no AI’ presentations respectively. However, in the AI diagnosis + segmentation format, 23 optometrists agreed with the reference standard and AI diagnosis, while seven diagnosed an ERM, likely prompted by small areas of epiretinal membrane (ERM) identified in the segmentation (Fig. 4).

#### Set 2

**Figure 5:**
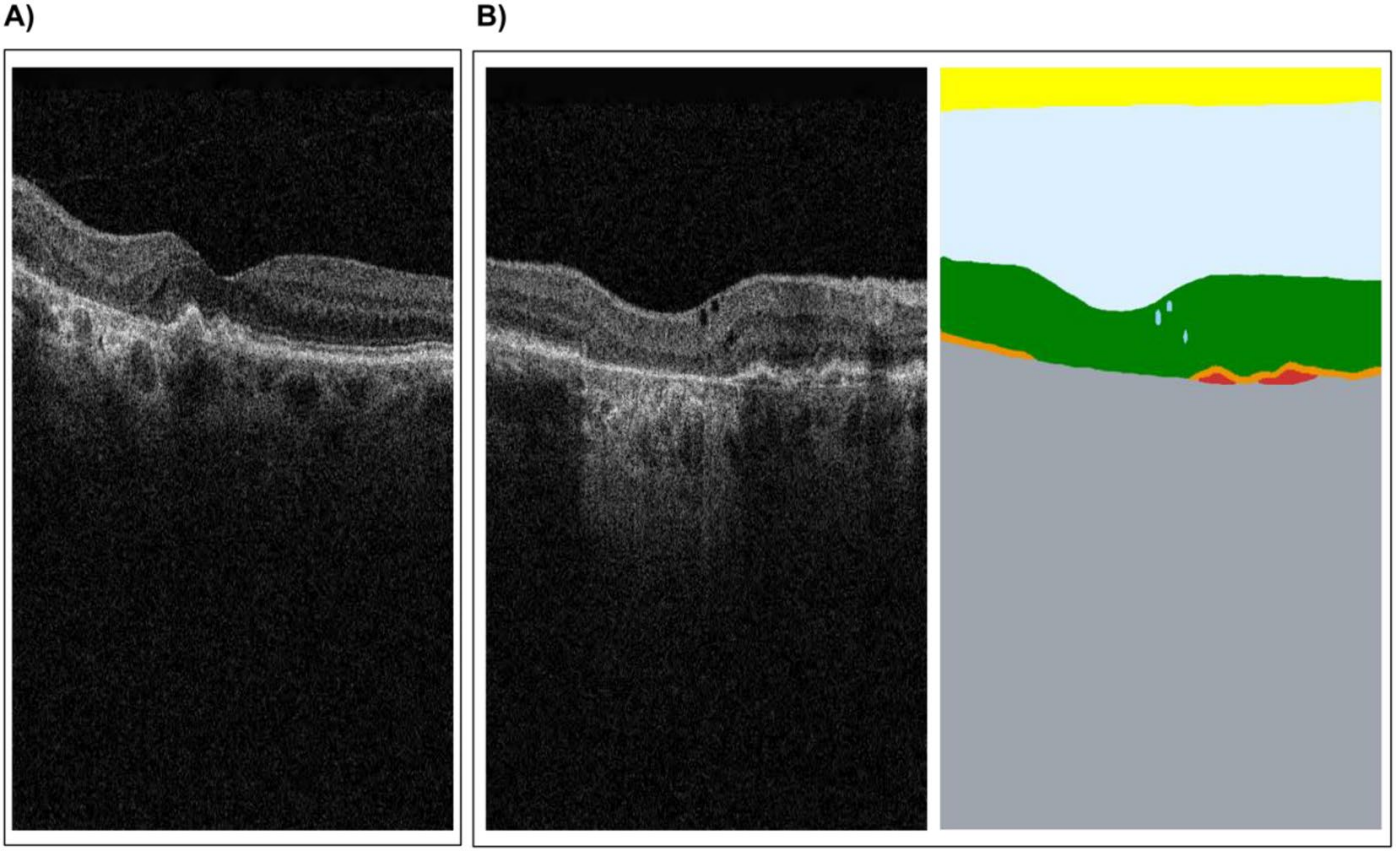
One image taken from two matched OCT scans. (**A**) OCT presented with AI diagnosis. (**B**) OCT presented with AI diagnosis plus segmentation. Similar areas of geographic atrophy with overlying minimal pockets of intra-retinal hypo-reflective spaces are present which for (**B**) were identified as intra-retinal fluid by the segmentation overlay (light blue pockets). In both cases there are adjacent PEDs to the atrophic areas, more marked in case (**A**). Both (**A**) and (**B**) were classified as having features of dry macular degeneration (geographic atrophy and drusen) by the AI diagnosis.

In this case the AI diagnosis was dry macular degeneration in agreement with the reference standard, which 29 participants also diagnosed for the ‘no AI’ and ‘AI diagnosis’ presentations. However, the segmentation identified possible areas of intra-retinal fluid overlying atrophy (corresponding to pseudocysts) and adjacent posterior epithelial detachment (PED) on the OCT, probably prompting 11 participants to diagnose the patient with choroidal neovascularisation (CNV, wet AMD) in the ‘AI diagnosis + segmentation’ presentation (19 diagnosed as dry AMD) (Fig. 5).

### Reported diagnostic confidence

**Figure 6:**
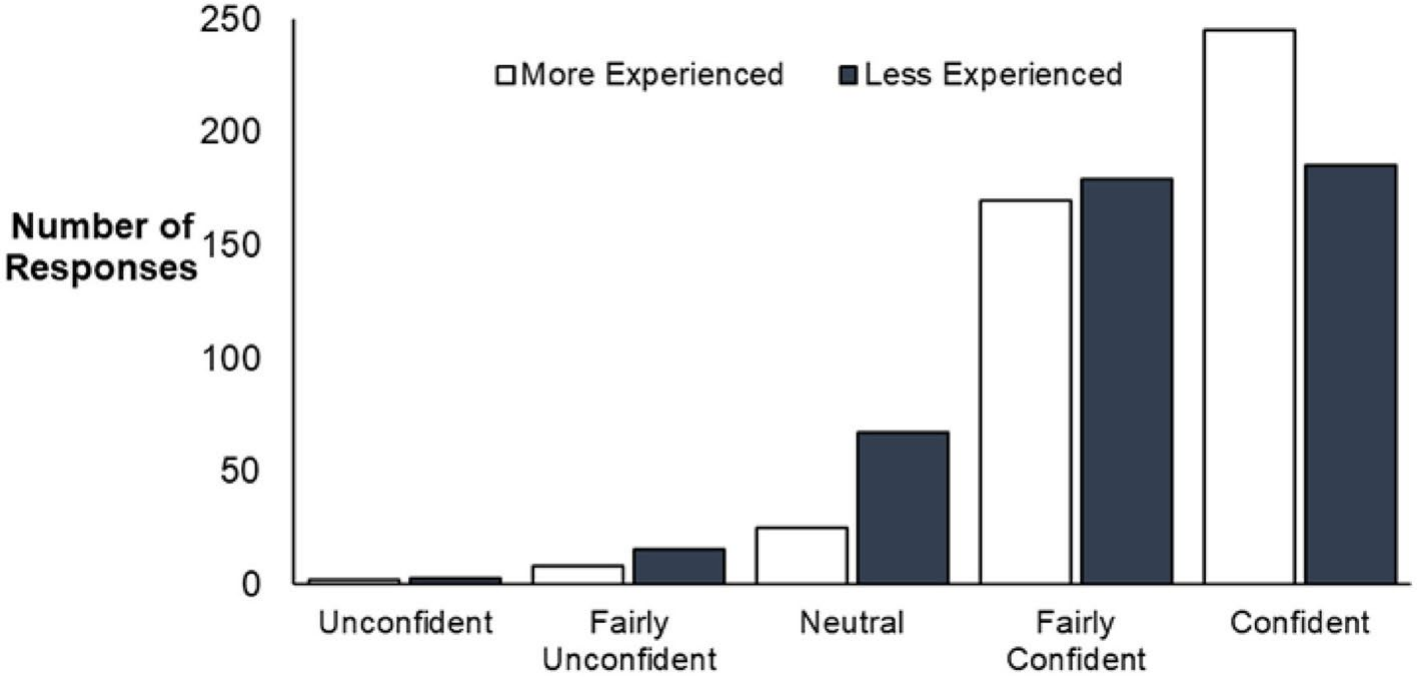
Total responses for diagnostic confidence (n = 900), divided into levels of experience (n = 450 more experienced, n = 450 less experiences). A significant difference in responses for confidence was found (p = 0.012) between the two groups based on experience, with more experienced participants overall more confident in their diagnostic decisions.

Overall, the more experienced participants were significantly more confident with their diagnoses than less experienced participants (p = 0.012) (Table 4, Fig. 6). No significant effect was found across the 3 groups based on presentation format (p = 0.461), order (p = 0.360) or any interaction between factors.

**Table 4:** Results from ANOVA testing on diagnostic confidence indicated by participants using a 5-point Likert scale.

Factor(s)	Confidence	
	F-value	p-value
1 Experience	7.429	0.0118*
2 Order	1.022	0.360
3 Presentation	0.774	0.461
4 Experience: Order	0.351	0.704
5 Experience: Presentation	1.315	0.269
6 Order: Presentation	1.014	0.406
7 Experience:Order:Presentation	0.902	0.468

ANOVA performed on results using aligned rank transform (ART). Results for factors 1–3 represent the effect of a single factor on diagnosis, confidence and trust. Results for factors 4–7 represent the effect of two or more factors interacting. Values in bold represent statistically significant results.

*p values considered statistically significant.

### Reported trust in AI

**Figure 7:**
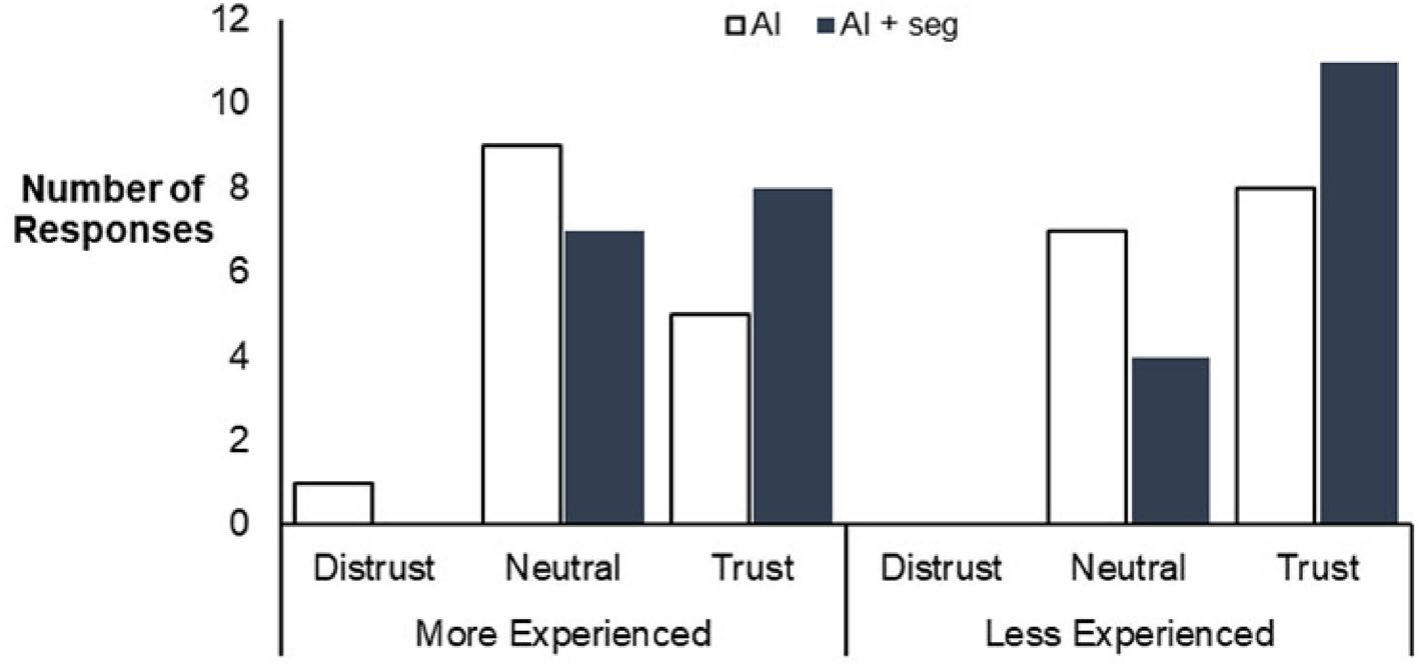
Total responses for level of trust (n = 60), divided into level of experience (n = 30 more experienced, n = 30 less experienced). A significant difference in responses for trust was found between the two groups based on experience (p = 0.038), with more experienced participants overall more confident in their diagnostic decisions. Significantly more participants trusted the AI plus segmentation overlays (AI + Seg) over the AI outputs alone (p = 0.029).

An ANOVA with ART adjustment revealed that participants trusted the AI significantly more when segmentation overlays were displayed compared to not (p = 0.029) (Table 5, Fig. 7). The less experienced participants reported a significantly higher level of trust compared to more experienced participants (p = 0.038). The case order had no significant effect on reported trust (p = 0.582). There was a significant interaction between level of experience and order (p = 0.049); however, there was no trend. No other significant interactions between factors were found.

**Table 5:** Results from ANOVA testing on level of trust in AI outputs indicated by participants using a 5-point Likert scale.

Factor(s)	Trust	
	F-value	p-value
1 Experience	4.842	0.038*
2 Order	0.548	0.582
3 Presentation	5.395	0.029*
4 Experience: Order	3.227	0.049*
5 Experience: Presentation	1.082	0.309
6 Order: Presentation	3.184	0.053
7 Experience: Order:Presentation	1.705	0.197

ANOVA performed on results using aligned rank transform (ART). Results for factors 1–3 represent the effect of a single factor on trust in AI. Results for factors 4–7 represent the effect of two or more factors interacting. Values in bold represent statistically significant results.

*p values considered statistically significant.

## Discussion

We explored the impact of introducing an AI-DSS on diagnostic decisions made by hospital optometrists when interpreting OCT scans. We expand on previous studies in other areas of medicine which have demonstrated a positive effect of human-AI collaboration when using a system of high diagnostic accuracy^4,26^; however, unlike previous work, we used a high proportion of cases (60%) in which the outputs of our AI system were incorrect (disagreed with the reference standard) or were ambiguous (more than one diagnosis proposed with high probability).

Overall, our participants made the most accurate diagnoses with respect to the reference standard when assessing the clinical cases without AI diagnostic support. This 'no Al' accuracy of 81% was very similar to the 80% mean diagnostic accuracy found by Jindal et al.^27^, where optometrists assessed retinal and optic nerve OCTs to determine whether either were 'diseased'.

The number of 'correct' responses decreased to 75% when AI diagnosis was presented in our cohort. We deliberately selected our cases based on AI outputs because, though infrequent, we aimed to explore how incorrect (whether stemming from a truly incorrect AI diagnosis or a disagreement with an imperfect reference standard) or uncertain AI diagnostic support may affect human diagnostic performance. The difference in practitioners’ responses aligning with the reference standard between the ‘no AI’ and ‘AI diagnosis’ presentations was of borderline significance and became non-significant when excluding the results from the three cases of ERM (supplementary material). A recent study by Tschandl et al.^4^ reported a negative effect of incorrect AI outputs on participants' diagnostic accuracy. That study, however, arbitrarily modified the output of an AI system to artificially produce incorrect results. We focussed instead on the (rare) actual cases where the AI system produced output inconsistent with the reference standard which does not automatically equate with incorrect output.

Even fewer diagnostic responses agreed with the reference standard when both AI diagnosis and AI segmentation were displayed (68%). The role of clinically ambiguous cases is likely to be the fundamental factor leading to this result. Cases where participants may have based their decisions on innocuous, subtle details revealed on the segmentation overlays rather than the AI diagnosis may offer an interesting and informative perspective on Human-AI interaction. Although the reference standard and the AI diagnosis were aligned in the examples identified, an alternative interpretation of the imaging in favour of an ERM being present (for set 1) and a CNV diagnosis (for set 2) could conceivably be made even by ophthalmology specialists.

These findings also highlight a conundrum on the value of presenting segmentation overlays to provide more information to clinicians, especially those less experienced in the interpretation of OCT scans. The diagnostic classification algorithm was trained on the segmentation produced by the segmentation algorithm; however, it was trained using clinical labelling of segmentations by experts at MEH, who were able to differentiate nuanced presentations of pathological OCT features highlighted by the segmentation algorithm in the broader context of each case. This creates different thresholds for pathology detection 'reference standards' and thus discrepancies between the segmentation and diagnostic outputs. For any AI systems in healthcare, a clear distinction is required between levels of ‘detectable’ and ‘clinically significant’ pathology and one must be careful when showing visualisations of intermediate stages to users, as they may be misinterpreted. Considering also the positive effect that the visualisations had on participants' trust, the effect of the segmentation overlays observed in our study suggests it is important for any additional visualisation to be aligned with the AI diagnostic output.

There were no significant differences between the number of correct responses from the two groups based on level of experience. This is contrary to findings of a previous study in ECG interpretation using a non-AI system^14^. However, we again compare our findings to Tschandl et al.^4^, whose diagnostic task was similar to ours, in that it used multi-class outputs and an AI-DSS. That study found an inverse relationship between the net gain from AI-based support and participant experience for an accurate AI system. Our combined findings suggest that less-experienced participants may benefit most from correct AI diagnostic support, but all users are equally influenced by incorrect outputs.

In our study, AI did not increase optometrists' diagnostic confidence, either with or without segmentation overlays. Bond et al.^14^ reported that incorrect automated diagnostic support significantly reduced interpreters' confidence. Despite our selection of 60% of cases where the AI was ‘incorrect’/ambiguous’ there was still no significant impact on diagnostic confidence for the full cohort. Future research should assess diagnostic confidence using the AI with its true diagnostic accuracy for clinical implementation^5^.

While AI in ophthalmology offers great potential, the social and legal challenges cannot be ignored. Reliability and accountability of the AI systems and their impact on clinical decision-making creates a complicated dynamic with healthcare professionals. For AI to be accepted by clinicians, both personally and institutionally, the systems must be reliable and trusted^28^. In this study, only one participant reported that they distrusted the AI diagnoses (without segmentation), with 16 neutral and 13 trusting. Given our case selection, it would have been possible to inadvertently introduce a bias against the system. Dietvorst et al.^29^, describe this as 'algorithm aversion', which is the reluctance to use algorithms known to be imperfect. Participants may detect the AI’s imperfect accuracy and uncertainty and calibrate their trust^30^ based on this isolated experience of using the AI.

Another challenge of introducing AI into clinical practice is the well-known "opaque box" problem^28^, describing many AI systems as non-transparent. Even though the accuracy of the AI was matched between the ‘AI diagnosis’ and ‘AI diagnosis plus segmentation’ presentations, the increased transparency with the segmentation overlays may have created the significantly higher level of trust in the AI when segmentations were displayed. This finding was particularly interesting in our study as although there was increased trust in the system when segmentations were displayed, participants agreed less on average with the AI diagnosis and reference standard in this presentation format (Table 3). Further research is required to explore how different elements of AI visualisations are utilised during clinical decision-making and which aspects most influence clinicians' OCT interpretation.

### Limitations

We have identified four main limitations to this study. Firstly, because the study was run remotely it was not possible to observe participants' decision-making processes. Future research with observations and/or detailed exit interviews would provide valuable insights into participants' interactions with AI systems. Although the remote set up allowed clinicians to complete the study at a time and pace that was convenient to them, it meant that we were unable to perform statistical analysis on the time taken for clinicians to review cases with and without AI support. This would also be an interesting focus for future study.

Secondly, the AI segmentation model was trained by human graders who annotated thousands of OCT slices for features of ocular pathology based on grading protocols. Such protocols mandated the annotation of any trace of features such as ERM even if not clinically significant. In such cases of trace ERM, both ‘ERM’ and ‘normal’ can be considered an acceptable diagnosis based on the different thresholds for detectable vs clinically significant pathology. In comparison, the reference standard clinical diagnosis would typically only diagnose pathology such as ERM if it was considered clinically significant. As a result, the classification of both AI and participant diagnostic decisions into ‘correct’ and ‘incorrect’ compared to the reference standard is occasionally ambiguous.

Our study involved matching across the three study conditions based on clinical case selection. Although our matched cases were confirmed by a medical retina specialist (KB), we recognise that individual cases are unique and that it would be impossible to find identical cases when matching for AI outputs, OCT appearance and clinical information.

Finally, while we aimed to maximise the ecological validity of the study, it was limited in both not reflecting a natural mix of cases and including less patient information than would normally be available.

## Conclusions

If AI support is to be adopted for the assessment of ophthalmological cases, it must be relied on appropriately. Our selection of cases, including an over-representation of cases where the AI-DSS was incorrect or uncertain compared to the reference standard, resulted in an interesting influence on diagnostic decisions made by optometrists from AI outputs of OCT scans irrespective of their level of experience. When segmentation overlays were presented, participants agreed with the reference standard the least. However, in some cases, AI segmentations highlighted true abnormal features, albeit innocuous and not necessitating medical attention, thus instigated disagreement with AI and reference standard diagnoses. It is not uncommon that the segmentation overlays highlight features of minimal clinical importance or force judgement calls on edge cases with nuanced clinical interpretation even by experts, which also puts into question the accuracy of the 'reference standard' in some of these cases. Despite reduced agreement, participants were inclined to trust the AI more when segmentations were displayed, perhaps since this renders the system more transparent.

In the field of Human–AI interaction, quantified analyses are valuable, yet the complexity of clinical practice and interpretation, the known imperfection of reference standards, and the distinction between detection of abnormal findings on imaging and clinically significant disease, demonstrate eloquently in this work that absolute conclusions cannot be drawn on the impact of AI-DSS on diagnostic performance of practitioners purely on the basis of quantified approaches. This offers useful directions for further mixed methods research to elucidate the thought processes and actual influence of AI-DSS as assisting technologies for clinicians.

### Supplementary Information

Supplementary Information.

## Data Availability

All of the de-identified participant data collected during the study will be linked to a data repository. Data queries should be directed to the corresponding author**.** The imaging data for the clinical cases were collected at Moorfields Eye Hospital NHS Foundation Trust and were provided in a deidentified format which is available only via the Moorfields Eye Hospital internal network. Data were used with both local and national permissions. This data is not available for sharing.
