# Supplementary material for: Diagnostic decisions of specialist optometrists exposed to ambiguous deep-learning outputs
Source: Sci Rep. 2024 Mar 21;14:6775. doi: 10.1038/s41598-024-55410-0 (PMC10958016; doi:10.1038/s41598-024-55410-0)
Supplement: Supplementary file 1 — Supplementary Information. [file 41598_2024_55410_MOESM1_ESM.pdf]

Supplementary Methods

Clinical Case Selection

The 30 cases were chosen to cover a range of macular pathologies, as well as to include healthy scans. When choosing cases, the diagnoses suggested by the AI were compared to the 'reference standard' diagnosis. The reference standard diagnosis was defined as each patient's clinical diagnosis, which was decided on by an ophthalmologist during the patient's visit to MEH. This involved an in-person examination with a full history and symptoms. For some patients, it may have involved additional diagnostic tests. The following categories were used to select cases. Full details of each matched set can be found in Supplementary Tables 1 and 2:

1. Normal (10% of cases) - For these cases, a normal OCT scan was displayed, which the AI classification algorithm correctly identified as normal.

2. Clear-cut (30% of cases) - For these cases, the diagnosis was 'clear-cut'. The cases clearly showed a diagnosis with no other suggestive findings of another diagnosis. This was clearly identified using the AI outputs and the segmentations.

3. False positive (10% of cases) - For these cases, the AI erroneously suggested an abnormality in a healthy retina.

4. Edge cases (40% of cases) - These were ambiguous cases. There may have been more than one possible diagnosis based on the information given.

5. False negative (10% of cases) - For these cases, the AI result identified a diagnosis that was less urgent than the reference standard diagnosis.

The cases were matched across the three types of presentation with respect to reference standard diagnosis and difficulty. The difficulty of the cases was matched through considering clinical information cues, OCT imaging and colour retinal photograph: i.e., how difficult each case would be to diagnose correctly without any AI support. The cases were purposely chosen to be difficult, thus included an artificially high number of situations where the AI was incorrect (false positives and negatives) or unsure (edge cases). This choice of cases was not revealed to participants until debriefing. This was to enable a focus on interesting cases whereby incorrect AI may influence participants' decisions.

Participant Experience

Thirty qualified optometrists were recruited to the study, all of whom currently worked within the hospital eye service. No minimum number of years' experience was required but optometrists had to be fully qualified. Participants were divided into two groups, based on

their level of experience in medical retina (MR) which was used as a surrogate for their familiarity of interpreting retinal OCT scans. The group allocation criteria are displayed in Supplementary Figure 1. If a participant was currently working in an MR clinic, and had been there for more than 1 year, they were allocated to the more experienced group. Others were allocated to the less experienced group, including those who had never worked in MR, who had not worked in MR in the past year, and those who had worked in MR for less than a year. This time period was decided with a consultant optometrist specialising in MR as most optometrists work in MR for only 1 or 2 sessions per week and require supervision for roughly the first 4-6 months. Also, without working in the clinic for over a year, OCT interpretation skills are likely to have degraded. It is acknowledged that this does not provide a distinct divide between more and less experienced groups, as optometrists may also have some knowledge of retinal OCT scans from outside MR clinics. However, these classification rules were chosen as a reasonable measure of level of experience.

Participant Training

Clear instructions were provided for how to navigate through the survey and how to clearly view the OCT volume scans prior to any study cases being presented. Participants were shown an example of an AI segmentation map along with the diagnosis probability percentages. This example was annotated with each aspect clearly explained. If the participant indicated that they were still unclear about what the AI segmentation and outputs represented, they were unable to complete the study at that point and were encouraged to contact the study investigator. All 30 participants indicated that they understood what the AI displayed. No information was given about the algorithms' diagnostic accuracy.

Participant Training - Segmentation Overlays

The following was shown to all participants during the training phase of the study:

You will also be provided with 'segmentation maps' produced using artificial intelligence (AI) algorithms. These maps display identified features within the OCT scan (for example intra-retinal fluid (IRF)). Segmentations are presented as overlays, covering the OCT scan. If a specific feature is identified, it is colour coded, based on a key that will be provided to you. An example can be seen below:

Example:

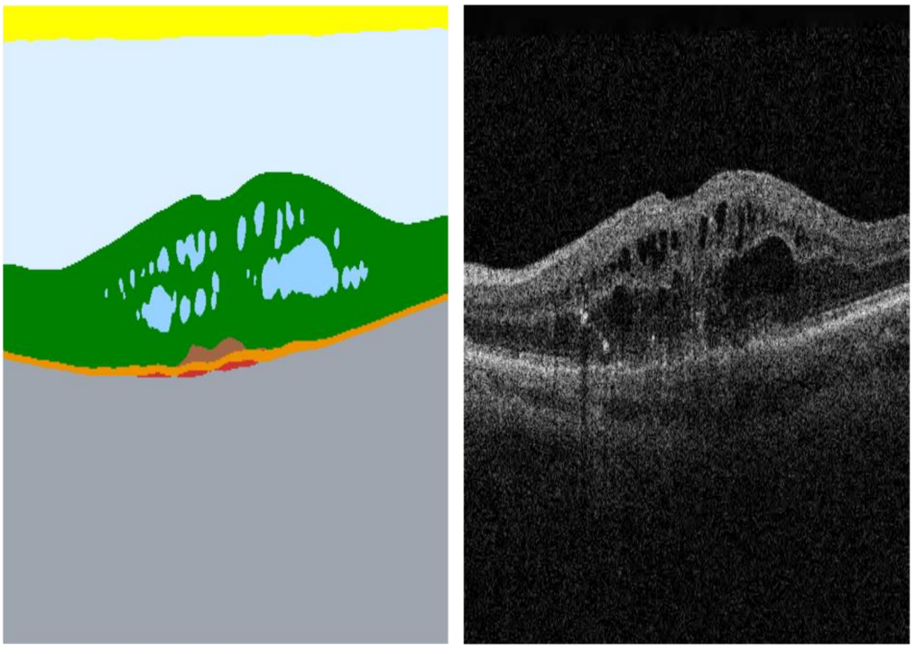

Key:

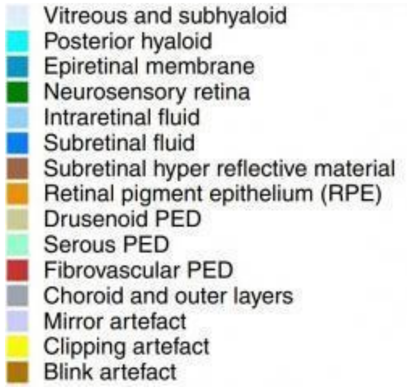

**(PED = Pigment epithelial detachment)

In this example, the segmentation has identified numerous large pockets of intra-retinal fluid. It has also identified a fibrovascular PED and sub-retinal hyper-reflective material. Other colour coded areas represent anatomical structures.

The results displayed in this segmentation map are then used by a separate AI algorithm to determine a suggested probable diagnosis.

Participant Training - AI Diagnostic Outputs

The following was shown to all participants during the training phase of the study:

You will also be provided with bar charts, presenting the output from an algorithm designed to suggest the most probable diagnosis as well as a referral suggestion. This algorithm uses

the results from the OCT segmentation maps to determine the most likely diagnosis or pathology present. The following image is an example of how this output will be presented:

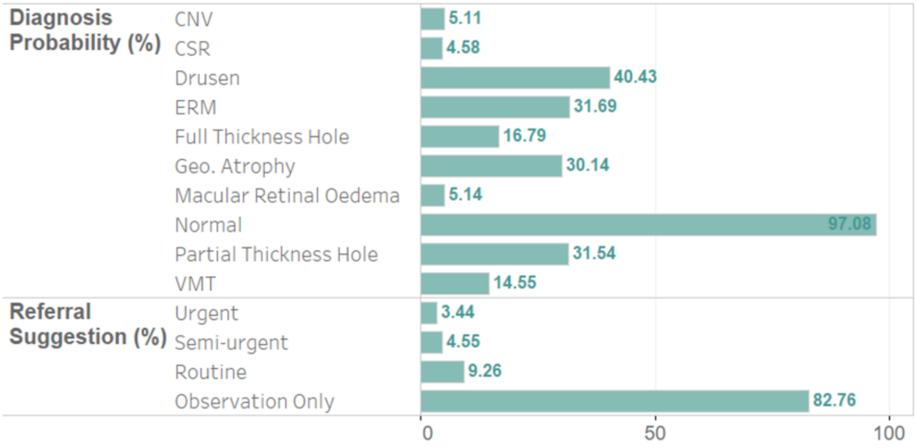

Each percentage is out of 100 and is the algorithm's output probability of each diagnosis being present. This example demonstrates a 97.08% probability that the OCT scan is normal.

The percentage for each diagnosis can be between 0-100%. The presence of each condition is assessed independently of the other diagnoses.

The AI may not always be as confident in its diagnosis. For example, consider the AI predicted a diagnosis of CNV with a value of 55% probability, but at the same time also predicted the diagnosis was MRO with 55%. For the two conditions considered independently, the AI predicts the same probability that both are present.

Statistical Methods

As our data did not meet the ANOVA assumptions, we used non-parametric tests for analysis. In particular we used the Aligned Rank Transform (ART) for factorial data, to assess the presence of interactions between N number of different factors. ART relies on a pre-processing step that aligns data before applying averaged ranks. After this step, common ANOVA and post-hoc analysis can be performed. By carrying out the pre-processing step, ART can be used in circumstances like the parametric ANOVA, despite the dependent variable being continuous or ordinal and not normally distributed. Appropriate post-hoc statistical comparisons with Bonferroni correction were used when ANOVA p values were significant between three or more groups.

Supplementary Exploratory Analysis

After running the analysis reported in the paper, we noticed that three cases across the conditions (n=1 'no AI', n=1 'AI diagnosis' and n=1 'AI diagnosis + segmentation') displayed very subtle epiretinal membranes on OCT imaging that would not be considered clinically significant. In order to assess whether our results for diagnostic accuracy and agreement with AI were significantly impacted by these three cases, we repeated the analysis excluding them. Thus, for each of the three case presentation formats, 270 diagnostic responses were assessed. An ANOVA with ART adjustment revealed significant differences in correct responses for the same factors as the original analysis; there was a significant difference across the three presentation formats (p<0·001) (Supplementary Table 1). A significant effect of the order of case presentation was again found (p=0·007). There was no significant effect of experience on the number of correct responses. When testing interactions between factors, a significant interaction between order and presentation (‘no AI’, ‘AI diagnosis’, ‘AI diagnosis + segmentation) was found (p=0·006). All other interactions showed no significant effect.

	**Diagnosis**	
**Factor(s)**	**F-value**	**p-value**
1 Experience	1.256	0.266
2 Order	5.38	**0.007***
3 Presentation	10.86	**<0.001**
4 Experience: Order	1.056	0.353
5 Experience: Presentation	2.166	0.122
6 Order: Presentation	3.926	**0.006***
7 Experience:Order:Presentation	0.523	0.719

* p values considered statistically significant

Supplementary Table 1: Results from ANOVA testing on number of correct diagnoses. ANOVA performed on results using aligned rank transform (ART). Results for factors 1-3 represent the effect of a single factor on diagnosis. Results for factors 4-7 represent the effect of two or more factors interacting. Values in bold represent statistically significant results.

Effect of presentation

The participants’ responses were divided into 3 classes, based on the presentation of information. In the ‘no AI’ group, 213/270 (79%) responses were correct. In the ‘AI diagnosis’ group, 196/270 (73%) were correct. In the ‘AI diagnosis + segmentation’ group, 181/270 (67%) were correct. Post-hoc testing with Bonferroni correction again revealed significant differences in correct responses between 2 pairs: no AI vs AI diagnosis + segmentation (p<0·001) and AI diagnosis + segmentation vs AI diagnosis (p=0·025). However, the differences between the no AI and AI diagnosis pairs were no longer significant (p=0·174).

This change from significant to non-significant is likely due to the smaller sample size creating less statistical power, as the difference in correct responses between these two conditions changed by just one response in the new analysis.

Participants' level of agreement with AI

We also assessed whether excluding the three cases affected the results for agreement with AI outputs with (AI diagnosis + segmentation) or without (AI diagnosis) segmentation overlays. The results again matched the original analysis whereby there was a significant effect of presentation format (p=0·006) (Supplementary Table 2) and no significant effect of experience (p=0·779) or order (p=0·822) or interaction effects.

	**Diagnosis**	
**Factor(s)**	**F-value**	**p-value**
1 Experience	0.08	0.779
2 Order	0.197	0.822
3 Presentation	8.15	**0.006***
4 Experience: Order	1.301	0.282
5 Experience: Presentation	0.883	0.352
6 Order: Presentation	0.195	0.824
7 Experience:Order:Presentation	0.407	0.668

* p values considered statistically significant

Supplementary Table 2: Results from ANOVA testing on number of responses in agreement with AI outputs. ANOVA performed on results using aligned rank transform (ART). Results for factors 1-3 represent the effect of a single factor on diagnosis. Results for factors 4-7 represent the effect of two or more factors interacting. Values in bold represent statistically significant results.

Supplementary Discussion

Effect of case order

Our study also revealed an effect of order on the number of correct diagnoses. In particular, the significantly higher number of correct ‘no AI’ responses when viewed third, in comparison to first, suggests that there was a learning effect during the study. Although different cases were viewed for different presentation formats, these cases were matched for disease type and difficulty. Participants may have been influenced by AI outputs seen prior to viewing the cases without AI output, despite being blinded to whether the AI was correct or incorrect. The potential of AI as a useful educational tool merits further evaluation.

Supplementary Figures

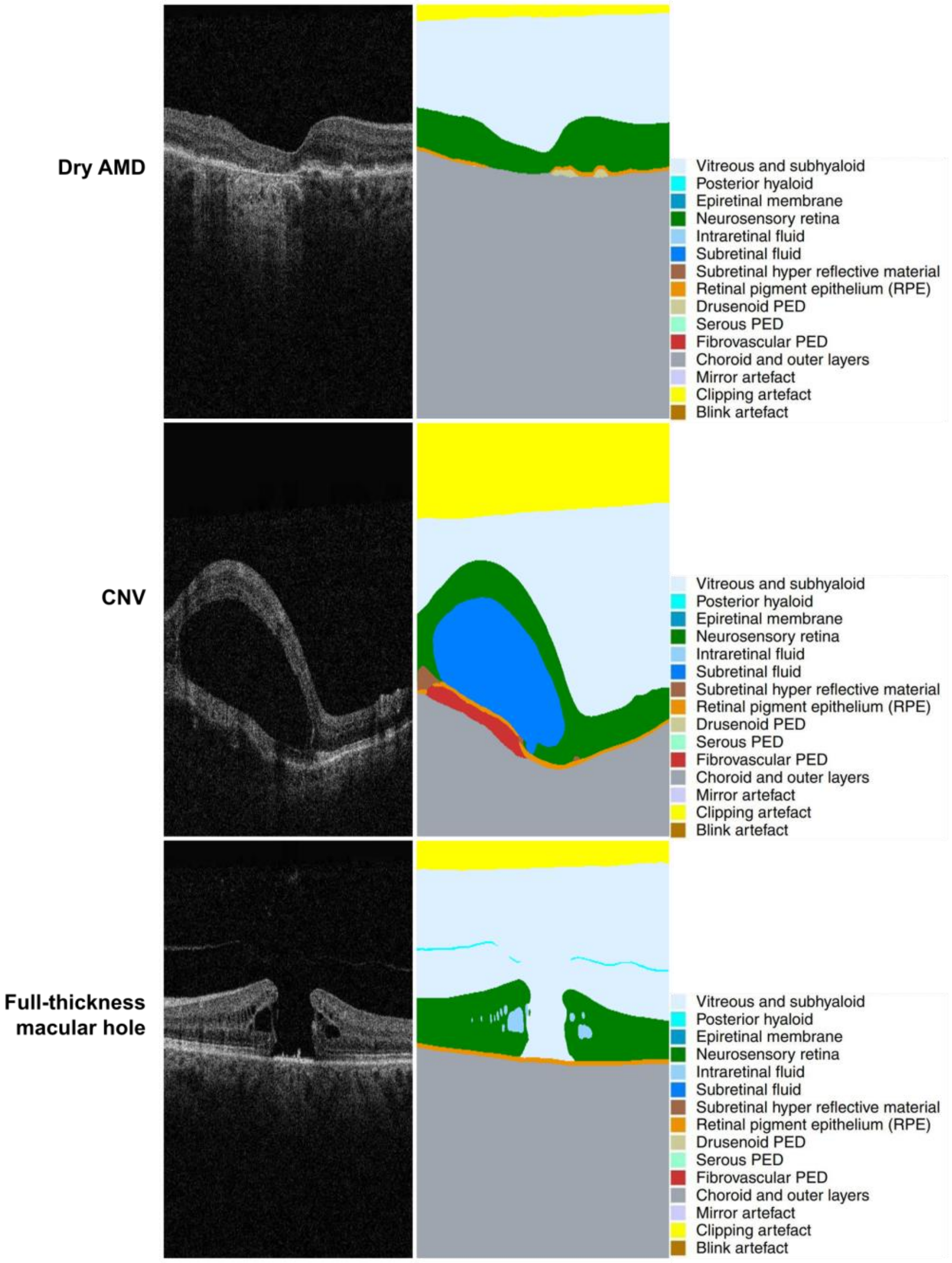

Supplementary Figure 1: Examples of OCT cases with corresponding segmentation overlays.

AMD = Age-related macular degeneration. CNV = choroidal neovascular membrane.

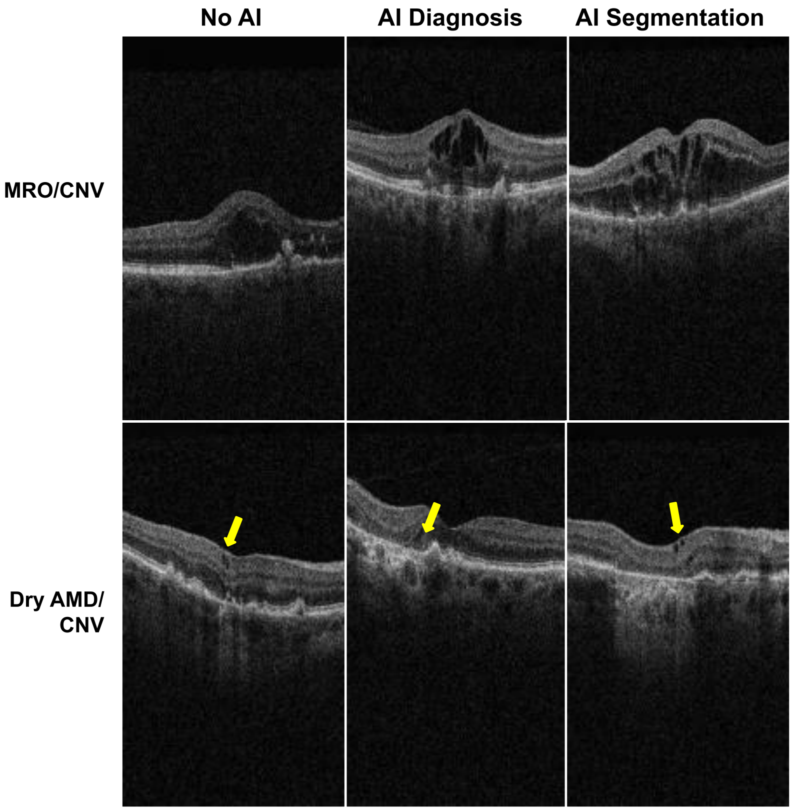

Supplementary Figure 2: Example of edge cases matched across the three conditions (arrows not displayed to participants but highlight regions of interest for the reader).

The choroidal neovascularization (CNV)/ macular retinal oedema (MRO) OCT images represent cases where there is substantial intra-retinal fluid (IRF) which could indicate either CNV or MRO. The CNV/ dry age-related macular degeneration (AMD) OCT images represent cases with small cysts overlying PEDs (yellow arrows).

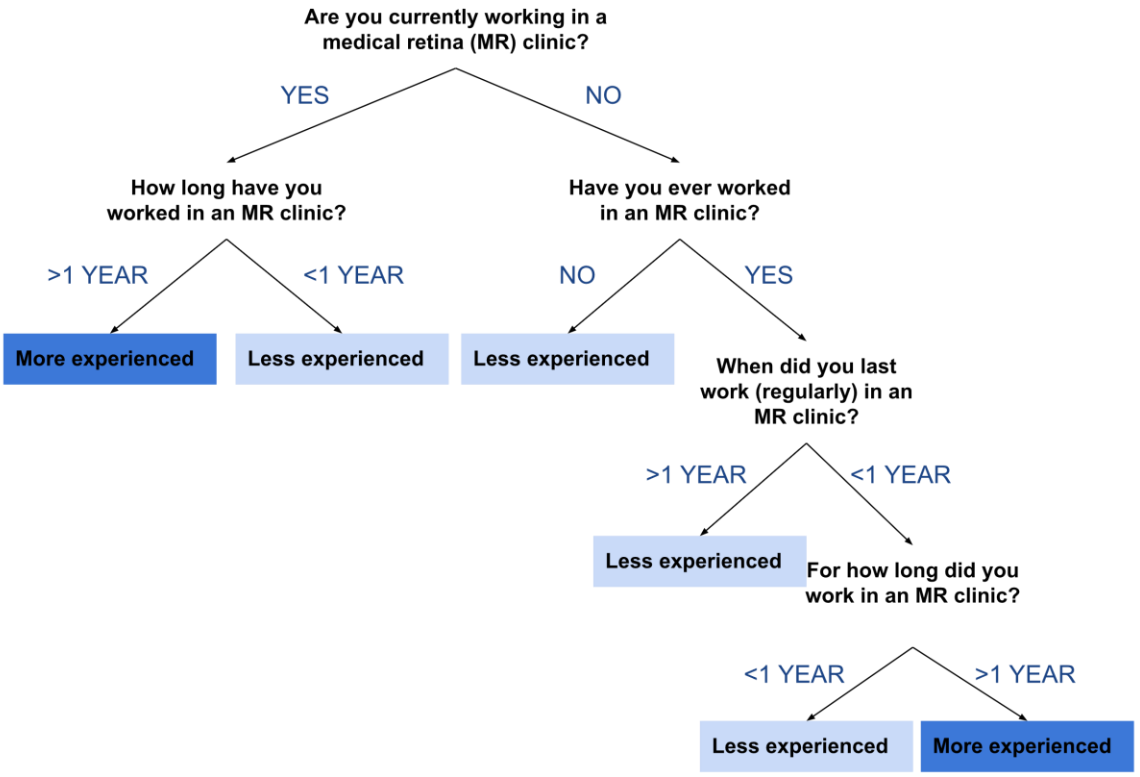

Supplementary Figure 3: The allocation of participants to one of two groups based on experience of OCT interpretation.

MR = Medical Retina

Supplementary Tables

Category	Set	Description
**Normal**	**1**	Clinical diagnosis is normal with no suspect pathology. AI output for normal is over 95% with no other diagnoses reaching over 50%.
**Clear Cut**	**2**	Clinical diagnosis is CNV with obvious features of CNV on both the retinal photograph (macular haemorrhaging) and OCT scan (significant intraretinal and/or sub retinal fluid with fibrovascular PED). AI output for CNV is over 95%.
	**3**	Clinical diagnosis is FTMH with obvious features of FTMH on both the retinal photograph (demarcated ring) and OCT scan. AI output for FTMH is over 99%.
	**4**	Clinical diagnosis is CSR with obvious features of CSR on the OCT scan (mainly a large, central dome-shaped area of SRF). AI output for CSR is over 90%.
**Edge Cases 'AI Diagnosis'**	**5**	Clinical diagnosis is normal with no suspect pathology on retinal photograph. OCT scan shows a small area of possible RPE irregularity. AI output for normal is over 50%. AI output for a feature of pathology is over 60%.
	**6**	Clinical diagnosis is CNV with minimal haemorrhaging on the retinal photograph. OCT scan shows significant intraretinal fluid. AI output for CNV is 70-90% and MRO 80-90% .
**Edge Cases 'AI Management'**	**7**	Clinical diagnosis is GA/drusen with no signs of active CNV on the retinal photograph. OCT scan additionally shows PEDs and cystic spaces. AI output for GA and drusen is over 99%. AI output for CNV is 40% or less. AI management suggestion 40-55% for both routine and urgent referral.
	**8**	Clinical diagnosis is GA/Dry AMD with significant areas of GA but no signs of active CNV on the retinal photograph. OCT scan additionally shows PEDs. AI output for GA and drusen is over 95%. AI output for CNV is 40% or less. AI management suggestion 40-55% for both routine and urgent referral.
**False Positive**	**9**	Clinical diagnosis is normal with no suspect pathology on the retinal photograph or OCT scan. AI output for normal is over 80%. AI output for VMT is over 50%
**False Negative**	**10**	Clinical diagnosis is CNV but with no clear signs of active CNV on retinal photograph. OCT scan shows small PEDs with minimal areas of overlying IRF. AI output for CNV is 20-30%.

Supplementary Table 3: Description of the 10 sets of cases matched across the three presentation formats to meet one of six categories. Edge cases (40%), false negatives (10%) and false positives (10%) were all chosen to be ambiguous. AI = Artificial Intelligence, CNV = Choroidal Neovascularisation, GA = Geographic Atrophy, IRF = Intra-retinal Fluid, FTMH = Full-Thickness Macular Hole, MRO = Macular Retinal Oedema, OCT = Ocular Coherence Tomography, PED = Pigment Epithelial Detachment, RPE =Retinal Pigment Epithelium, SRF = Sub-retinal Fluid, VMT = Vitreo-macular Traction.

		Clinical Information					AI Referral Suggestion Outputs				AI Diagnostic Suggestion Outputs									
Category	Set	Presentation	Age	Sex	VA	Clinical Diagnosis	Urgent	Semi-urgent	Routine	Observe	Normal	MRO	CNV	Drusen	GA	CSR	FTMH	PTMH	VMT	ERM
Normal	1	No AI	49	M	6/6	Normal	-	-	-	-	-	-	-	-	-	-	-	-	-	-
		AI	33	F	6/6	Normal	0.028	0.041	0.122	0.810	0.965	0.041	0.037	0.359	0.327	0.037	0.160	0.310	0.155	0.281
		AI + Segmentation	33	F	6/6	Normal	0.033	0.054	0.134	0.779	0.955	0.046	0.040	0.390	0.329	0.045	0.172	0.312	0.125	0.395
Clear Cut	2	No AI	66	M	6/15	CNV	-	-	-	-	-	-	-	-	-	-	-	-	-	-
		AI	80	M	6/30	CNV	0.982	0.008	0.007	0.004	0.064	0.026	0.980	0.592	0.502	0.038	0.119	0.414	0.347	0.328
		AI + Segmentation	95	M	6/36	CNV	0.968	0.011	0.017	0.005	0.096	0.036	0.962	0.660	0.304	0.149	0.091	0.440	0.309	0.297
	3	No AI	84	M	3/60	FTMH	-	-	-	-	-	-	-	-	-	-	-	-	-	-
		AI	68	M	6/60	FTMH	0.010	0.031	0.950	0.010	0.079	0.036	0.020	0.599	0.194	0.035	0.992	0.247	0.382	0.666
		AI + Segmentation	69	F	6/60	FTMH	0.012	0.005	0.979	0.004	0.059	0.008	0.027	0.587	0.459	0.030	0.998	0.260	0.602	0.675
	4	No AI	36	M	6/18	CSR	-	-	-	-	-	-	-	-	-	-	-	-	-	-
		AI	67	M	6/6	CSR	0.556	0.015	0.422	0.007	0.069	0.025	0.494	0.606	0.436	0.977	0.146	0.292	0.186	0.253
		AI + Segmentation	43	M	6/12	CSR	0.119	0.023	0.851	0.007	0.070	0.031	0.093	0.364	0.325	0.942	0.163	0.312	0.197	0.165
Edge Cases 'AI Diagnosis'	5	No AI	64	F	6/9	Normal	-	-	-	-	-	-	-	-	-	-	-	-	-	-
		AI	81	F	6/12	Normal	0.151	0.053	0.572	0.223	0.636	0.040	0.114	0.917	0.284	0.055	0.224	0.340	0.481	0.182
		AI + Segmentation	55	F	6/9	Normal	0.183	0.087	0.578	0.151	0.520	0.067	0.133	0.372	0.245	0.681	0.162	0.287	0.316	0.150
	6	No AI	83	M	6/12	CNV	-	-	-	-	-	-	-	-	-	-	-	-	-	-
		AI	56	F	6/18	CNV	0.617	0.347	0.034	0.003	0.027	0.884	0.777	0.937	0.361	0.096	0.148	0.275	0.349	0.207
		AI + Segmentation	80	F	6/15	CNV	0.565	0.395	0.034	0.006	0.055	0.903	0.765	0.963	0.438	0.116	0.199	0.283	0.145	0.349
Edge Cases 'AI Management'	7	No AI	87	F	6/60	GA	-	-	-	-	-	-	-	-	-	-	-	-	-	-
		AI	85	F	6/9	GA	0.499	0.019	0.476	0.006	0.071	0.025	0.258	0.997	0.999	0.040	0.146	0.334	0.305	0.890
		AI + Segmentation	90	F	6/18	GA	0.550	0.021	0.423	0.006	0.065	0.027	0.402	0.990	0.997	0.031	0.140	0.342	0.236	0.659
	8	No AI	70	M	6/24	GA	-	-	-	-	-	-	-	-	-	-	-	-	-	-
		AI	92	F	6/18	GA	0.470	0.048	0.471	0.011	0.088	0.071	0.392	0.994	0.959	0.031	0.155	0.319	0.476	0.404
		AI + Segmentation	70	M	6/60	GA	0.434	0.018	0.543	0.005	0.059	0.021	0.169	0.998	0.999	0.033	0.104	0.340	0.135	0.757
False Positive	9	No AI	74	F	6/6	Normal	-	-	-	-	-	-	-	-	-	-	-	-	-	-
		AI	68	M	6/6	Normal	0.031	0.020	0.130	0.819	0.965	0.030	0.049	0.485	0.232	0.027	0.220	0.371	0.504	0.201
		AI + Segmentation	29	M	6/6	Normal	0.092	0.069	0.352	0.487	0.855	0.065	0.076	0.491	0.359	0.029	0.222	0.384	0.683	0.292
False Negative	10	No AI	34	F	6/12	CNV	-	-	-	-	-	-	-	-	-	-	-	-	-	-
		AI	36	M	6/12	CNV	0.289	0.073	0.582	0.056	0.298	0.065	0.234	0.832	0.304	0.341	0.190	0.313	0.271	0.315
		AI + Segmentation	81	M	6/10	CNV	0.348	0.123	0.514	0.015	0.077	0.124	0.263	0.998	0.969	0.028	0.197	0.294	0.278	0.513

Supplementary Table 4: Clinical information and raw AI diagnostic and referrals outputs (to 3dp) for the cases shown in the three presentation formats: No AI, AI and AI plus segmentation. Each output is out of a maximum of 1 and was converted to a percentage when displayed to participants. The management suggestions highlighted in orange show the AI's suggested management. The diagnostic suggested highlighted in green show the diagnoses determined as 'present' by the AI, i.e., a score of over 0.50. CNV = Choroidal Neovascularisation, CSR = Central Serous Retinopathy, ERM = Epiretinal Membrane, GA = Geographic Atrophy, FTMH = Full-Thickness Macular Hole, MRO = Macular Retinal Oedema, PED = Pigment Epithelial Detachment, PTMH = Partial Thickness Macular Hole, RPE =Retinal Pigment Epithelium, SRF = Sub-retinal Fluid, VMT = Vitreo-macular Traction.
